# Surgical or Traumatic Rheumatism

**Published:** 1904-07-30

**Authors:** 


### SURGICAL OR TRAUMATIC RHEUMATISM.

THIS term is applied by Dr. Jas. Weir[1] to a condition characterised by aching pain and stiffness in a joint, muscle, or tendon, influenced as a rule by atmospheric conditions, and following, it may be after a considerable interval, some form of local violence. Dr. Weir's argument is that whilst in some of these cases a constitutional cause may have to be recognised and treated, the most important therapeutic measure is the use of Corrigan's button. He has met with numerous examples of this condition in which, though a long course of internal medication had proved of no avail, the cautery gave prompt relief. He has had a similar experience in cases of lumbago which had been unsuccessfully treated by high frequency currents.

[1] Glasgow Med. Jour., July 1904.

